# A Blood Flow Volume Linear Inversion Model Based on Electromagnetic Sensor for Predicting the Rate of Arterial Stenosis

**DOI:** 10.3390/s19133006

**Published:** 2019-07-08

**Authors:** Dan Yang, Yan-jun Liu, Bin Xu, Yun-hui Duo

**Affiliations:** 1 College of Information Science and Engineering, Northeastern University, Shenyang 110819, China; 2 Key Laboratory of Data Analytics and Optimization for Smart Industry, Northeastern University, Shenyang 110819, China; 3 College of Computer Science and Engineering, Northeastern University, Shenyang 110819, China

**Keywords:** electromagnetic sensor, artery stenosis, COMSOL, finite element, weight function

## Abstract

This paper presents a mathematical model of measuring blood flow based on electromagnetic induction for predicting the rate of arterial stenosis. Firstly, an electrode sensor was used to collect the induced potential differences from human skin surface in a uniform magnetic field. Then, the inversion matrix was constructed by the weight function theory and finite element method. Next, the blood flow volume inversion model was constructed by combining the induction potential differences and inversion matrix. Finally, the rate of arterial stenosis was predicted based on mathematical relationship between blood flow and the area of arterial stenosis. To verify the accuracy of the model, a uniform magnetic field distribution of Helmholtz coil and a 3D geometric model of the ulnar artery of the forearm with different rates of stenosis were established in COMSOL, a finite element analysis software. Simulation results showed that the inversion model had high accuracy in the measurement of blood flow and the prediction of rate of stenosis, and is of great significance for the early diagnosis of arterial stenosis and other vessel diseases.

## 1. Introduction

Blood is an important carrier of oxygen and nutrients between the organs and tissues of the body. Arterial stenosis will reduce the blood flow to various organs or tissues, causing coronary heart disease, angina, and other cardiovascular diseases. According to the statistics of the World Health Organization, 17 million people died of cardiovascular diseases in 2008, accounting for 30% of the global total [1]. Atherosclerosis is one of the main causes of arterial stenosis, which is characterized by the blockage of blood vessels by the plaques formed by the accumulation of lipids. The best treatment methods for vessels with different degrees of stenosis are also different. Therefore, it is of great value to develop a safe and non-invasive method to monitor arterial vessel blood flow status for common arterial vessel diseases early prevention.

Nowadays, common diagnostic methods for arterial stenosis include digital subtraction angiography (DSA), nuclear magnetic resonance angiography (MRA), spiral CT angiography (CTA), and ultrasonic examination. DSA has a high spatial resolution and can accurately detect the degree and range of arterial stenosis [2]. However, due to its high trauma and high diagnostic cost, DSA is generally used as the examination before angioplasty, rather than suitable for general survey [3]. MRA is a non-invasive angiography technique, which has a good consistency with the results of DSA detection, but the arterial stenosis shown by MRA may be exaggerated [4]. CTA can obtain the complete morphology of complex structures and show calcified plaques [5]. When the stenosis degree is large, the results can be consistent with DSA detection [6]. However, patients need to be injected with contrast media and the method is radioactive [7]. Compared with DSA, MRA and CTA, the ultrasonic diagnosis of arterial stenosis has the advantages of non-trauma and convenience [8]. However, the disadvantage of this method lies in the scattering and echo of ultrasonic signals in human body propagation, which brings certain difficulty to the measurement and relies on the rich experience of operators [9], because the diagnostic accuracy of this method is affected by the Doppler angle of maladjustment, the position and size of the sampling body, the pulse repetition frequency, and the gain setting and other device parameters [10,11]. In addition, MRA and CTA will produce harmful x-rays to physicians, and excessive radiation can damage the activity of human cells [12]. Therefore, it is urgent to find a diagnostic device and method that is harmless to doctors, convenient to use, and takes into account the advantages of existing technologies.

As is known to all, there is an inverse mathematical relationship between blood flow and arterial sectional area, so it is feasible to predict arterial stenosis by measuring blood flow. Traditional blood flow measurement methods include injection tracer method, volume method, impedance method, and the thermal dilution method [12,13,14,15]. The accuracy of these detection methods is low, the operation is complex, and will cause damage to the human body. At present, the commonly used clinical methods include ultrasonic Doppler method, magnetic resonance imaging method, etc. The Ultrasonic Doppler method is to use the Doppler effect of ultrasound to measure blood flow velocity [16]. However, the disadvantage of this method lies in the scattering and echo of ultrasonic signals in the propagation of human body, which brings certain difficulties to the measurement [17]. Magnetic resonance imaging is an imaging technology that measures the velocity of liquid flow by using the phase change of proton signal collected by magnetic resonance due to liquid flow [18]. However, it has its own shortcomings, such as slow imaging speed, and the acquisition of high-quality images depends on the rich experience of operators [19].

Recently, many researchers have made some progress in measuring the flow of uniformly conductive liquid based on electromagnetic induction. Raymond O. Webilor ’work described a novel dual-frequency inductive flow tomography (IFT) system, which relied on the use of a multi-electrode electromagnetic flow meter (EMFM). This flow meter is currently capable of imaging the velocity profile of the conducting continuous phase of both single phase and highly asymmetric multiphase flows ten times every second [20]. László E Kollár’ work improved the previous velocity profile reconstruction method and made it suitable for both axisymmetric and asymmetric flows [21]. X. Li’ work presented a novel finite element procedure for the solution of the electromagnetic flow meter weight function [22]. In H A Abdul Wahhab’ work, electromagnetic induction technique of measuring void fraction in liquid/gas fuel flow was utilized. The results had been proven that the electromagnetic induction is a feasible technique for the actual measurement of void fraction in a Diesel/CNG (Compressed Natural Gas) fuel flow [23]. Current technologies for measuring fluids based on electromagnetic induction make it possible to measure human blood flow.

In view of the shortcomings of existing blood flow measurement methods, this paper proposed a non-invasive blood flow measurement inversion model based on electromagnetic induction. The model combined the characteristics of blood flow with the principle of electromagnetic induction. In a uniform magnetic field, blood flow will create a steady field of induced potential around the blood vessels. Multiple groups of induced potential differences can be collected from the human skin surface by pairs of electrode sensors. The blood flow measurement matrix was established by using weight function theory and finite element method. Finally, the rate of arterial stenosis was predicted based on the mathematical relationship between blood flow and arterial cross-sectional area. This theoretical measurement model based on electromagnetic sensor to detect the rate of arterial stenosis is not affected by the uneven velocity profile and can predict the rate of arterial stenosis in a non-invasive and economical way. In the future, it can be developed into a beneficial, more convenient for physicians, and non-invasive for patients method for the diagnosis of arterial stenosis.

## 2. Theory of the Electromagnetic Induction

**Figure 1:**
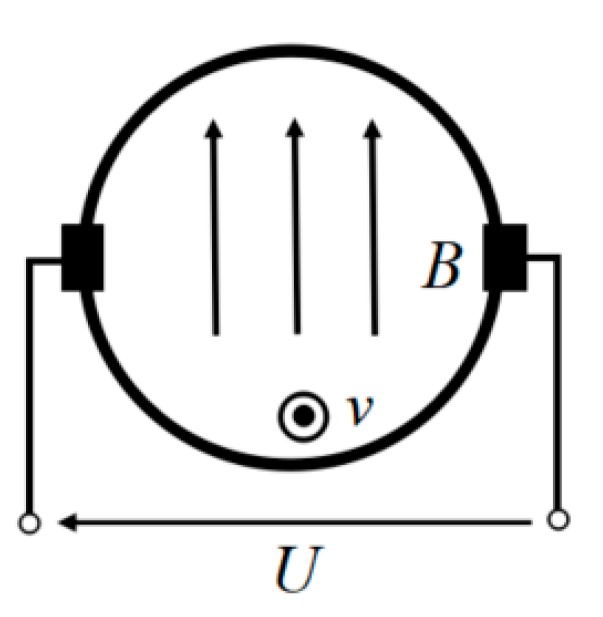
The schematic diagram of induced potential.

According to Faraday’s law of induction [24], when a conducting fluid moves through a uniform and static magnetic field B with a velocity v as shown in Figure 1, the charged particles of the fluid experience a force called the Lorentz force. In this case, the electric current density J in the conducting fluid, in the presence of electric and magnetic fields, is given by Ohm’s law:

(1) $$J=\sigma \left(E_{C}+v\times B\right)$$

where σ is the local fluid conductivity, $E_{C}$ is electrostatic field, The expression (v×B) represents the local induced electric field induced by the fluid motion.

According to the relevant theories of electromagnetic induction [24,25,26,27] and assuming that the conductivity in the flow section is uniform, we can obtain the general partial differential equation (Laplace equation) to measure the electromagnetic induced potential of the fluid with uniform conductivity:

(2) $$\nabla^{2}U=\nabla \cdot \left(v\times B\right)$$

Solving this equation, by the application of the appropriate boundary conditions, gives the electrical potential distribution U due to the motion of the fluid in the uniform magnetic field B. Hence, the blood flow information can be reconstructed by obtaining the induction electric potential change.

## 3. Mathematical Model

### 3.1. Mathematical Model of Induction Potential Difference

The traditional electromagnetic induction calculation method has a large error when the blood flow velocity of human artery is non-axisymmetric in the flow profile. Based on the weight function theory proposed by Shercliff [28] and the finite element analysis method, the three-dimensional mathematical model of flow induced potential difference proposed by Bevir [29] is simplified into a two-dimensional model in this paper:

(3) $$\Delta U=\int_{0}^{2\pi }\int_{0}^{R}W\left(r,\theta \right)\cdot v\left(r,\theta \right)\cdot rdrd\theta$$

where ΔU is the flow induction potential difference, R is the measured section radius, (r,θ) represents the flow position, W(r,θ) represents the weight function value of each point on the measured section, and v(r,θ) represents the velocity value of each axial point. Since the radius of the human artery is small, the information of the center of the artery section can be approximately expressed as the average information of the entire artery section, and Equation (3) can be further simplified as:

(4) ΔU=W(r,θ)⋅v(r,θ)⋅A

A is the cross section area of the artery, and the stenosis of the artery will directly reduce the cross section area, thus reducing the induction potential difference. Equation (4) is the theoretical calculation model of induction potential difference. By solving the weight function value of each point, the theoretical value of induction potential difference can be obtained.

### 3.2. Mathematical Model of Weight Function

Based on Bevir’s three-dimensional mathematical model, the virtual current density vector $J_{v}$ [30] is introduced into the weight function, which is defined as follows:

(5) $$W=B\times J_{v}$$

where B is the magnetic flux density vector, and suppose that the magnetic field only has a non-zero component along the y-axis, that is $B=\left[0,B_{0},0\right]$. Then, the mathematical model of virtual current density vector $J_{v}$ was derived by Laplace equation:

(6) $$J_{v}=\left[\begin{array}{c} J_{r}\\ J_{\theta } \end{array}\right]=\left[\begin{array}{c} \frac{1}{R\pi }\sum_{n=1}^{\infty }{\left(\frac{r}{R}\right)}^{n-1}\left(\cos n\left(\psi_{out}-\theta \right)-\cos n\left(\psi_{in}-\theta \right)\right)\\ \frac{1}{R\pi }\sum_{n=1}^{\infty }{\left(\frac{r}{R}\right)}^{n-1}\left(\sin n\left(\psi_{out}-\theta \right)-\sin n\left(\psi_{in}-\theta \right)\right) \end{array}\right]$$

Combined with Equations (5) and (6), the weight function only has a unique non-zero component along the z-axis, and its mathematical model is as follows:

(7) $$W\left(r,\theta \right)=\frac{B_{0}}{R\pi }{\sum_{n=1}^{\infty }\left(\frac{r}{R}\right)}^{n-1}[\cos\left(n\psi_{out}-\left(n-1\right)\theta \right)-\cos\left(n\psi_{in}-\left(n-1\right)\theta \right)]$$

where $\psi_{in}$ and $\psi_{out}$, respectively, represent the virtual current inflow and outflow angle, namely the polar coordinate angle of the measured potential and the reference potential in the actual measurement. By selecting different values of $\psi_{in}$ and $\psi_{out}$, multiple groups of induced potential differences can be obtained. From the above equation, it can be seen that the weight function value is only related to the size of magnetic field, measured section size and measured potential position, and has nothing to do with the inherent characteristics of the fluid.

### 3.3. Blood Flow Volume Inversion Model

**Figure 2:**
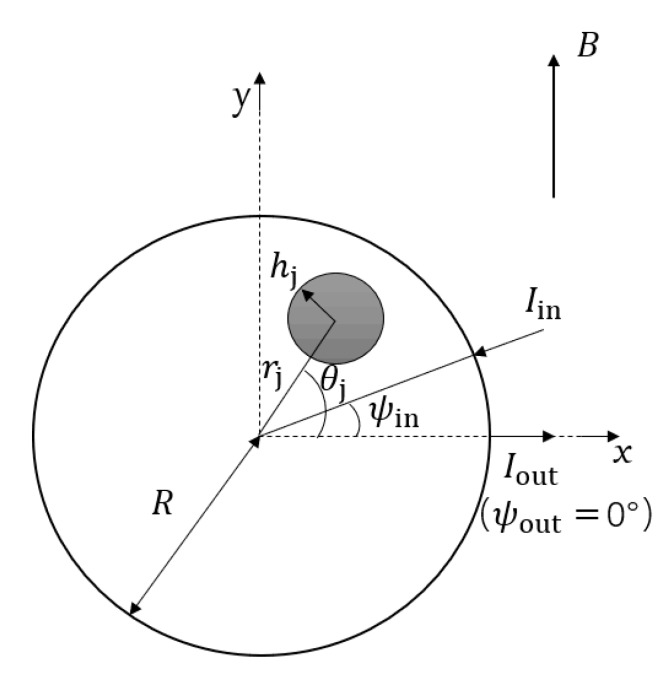
The schematic diagram of arterial blood flow measurement principle.

The schematic diagram of arterial blood flow measurement principle is shown in Figure 2. The artery is approximately viewed as a small channel within the measured section, and the blood flow Q through the measured section has a linear relationship with the area:

(8) $$Q=v\cdot A=v\cdot \pi \cdot h_{j}^{2}$$

Substitute Equation (8) into Equation (4) to obtain the basic formula for arterial blood flow inversion:

(9) $$Q_{j}=W_{ij}{}^{-1}\cdot \Delta U_{i}$$

where $\Delta U_{i}$ is the flow induction potential difference at the $i^{th}$ electrode, $W_{ij}$ represents the weight function of the area of the $j^{th}$ artery at the $i^{th}$ electrode, and $Q_{j}$ is the flow rate of the $j^{th}$ artery.

Assuming that there are i+1 electrodes and j arteries for the actual measurement, Equation (9) is converted into a multidimensional matrix form for analysis and calculation:

(10) $$\left(\begin{array}{c} Q_{1}\\ \vdots \\ Q_{j} \end{array}\right)={\left(\begin{array}{ccc} W_{11} & \cdots & W_{1j}\\ \vdots & \ddots & \vdots \\ W_{i1} & \cdots & W_{ij} \end{array}\right)}^{-1}\left(\begin{array}{c} \Delta U_{1}\\ \vdots \\ \Delta U_{i} \end{array}\right)$$

or:

(11) $$Q=W^{-1}U$$

Equation (11) is the blood flow volume inversion model. Where U is the input vector, Q is the output vector, and $W^{-1}$ is the inversion matrix composed of i×j weight function values. But in fact that W is an approximate singular matrix, the direct inverse calculation results have a large error. In this paper, the inversion of W was performed using a Tikhonov regularization technique involving singular value decomposition (SVD) of W.

The arterial blood flow information can be reconstructed through Equation (11), and the degree of arterial stenosis can be approximately predicted by comparing the arterial blood flow with the normal arterial blood flow, and the stenosis rate can be calculated as follows:

(12) $$\delta_{j}=\left(1-\frac{Q_{j}}{Q_{0}}\right)\times 100\%$$

where $Q_{j}$ is the blood flow value of the $j^{th}$ artery, $Q_{0}$ is the normal arterial blood flow, and $\delta_{j}$ is the stenosis rate of the $j^{th}$ artery.

In summary, if the arterial stenosis changes, the arterial blood flow will change, then, the electromagnetic induction potential will change in a uniform magnetic field. Therefore, we can obtain the inductive potential difference by measuring, and calculate the arterial blood flow information by the blood flow inversion model. Finally, we can predict the rate of arterial stenosis accurately by the mathematical relationship between blood flow and the area of arterial stenosis.

## 4. Simulation Results and Analysis

### 4.1. Simulation Model of Blood Flow Potential Difference Measurement

**Figure 3:**
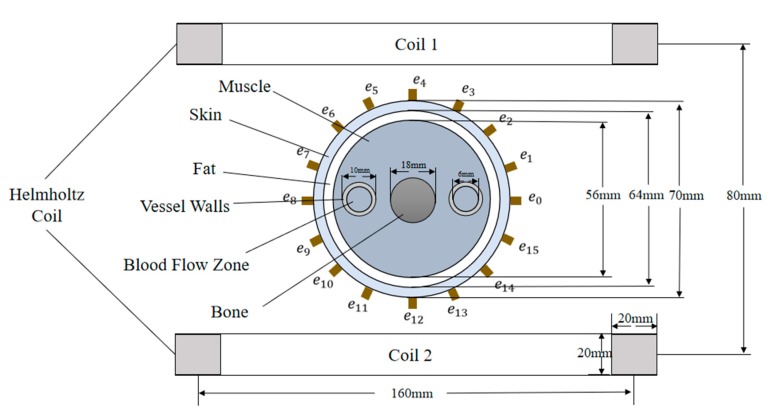
The basic structure of electromagnetic sensor used to predict arterial stenosis rate.

Before the formal simulation experiment, we designed an electromagnetic sensor structure for detecting arterial blood flow and predicting the stenosis rate of upper arm artery based on the theories in Section 2 and Section 3, as shown in Figure 3.

Figure 3 mainly includes an electromagnetic sensor and cross section of upper arm of human body. Electromagnetic sensors consist mainly of a set of Helmholtz Coils used to generate a uniform magnetic field and 16 electrodes (e_0_, e_1_ ... e_15_) used to detect potential differences in the surface of the skin. The cross section of the upper arm shows the basic components of the upper arm, including skin, fat, muscle, blood vessel wall, blood vessel and bone. The dimensions and positions of these physiological tissues adopted in the simulation have been indicated in Figure 3, and the approximate true value of each tissue size is adopted [31].

**Figure 4:**
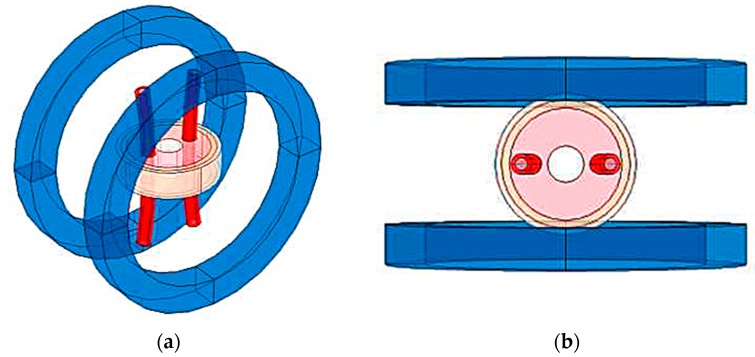
The basic model of simulation measurement: (**a**) 3D view; (**b**) X-Y plane view.

According to Figure 3, we established a 3D simulation model in COMSOL Multiphysics, as shown in Figure 4.

**Figure 5:**
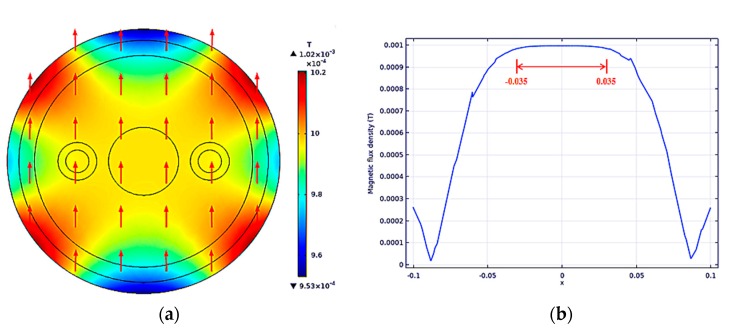
The uniform magnetic field distribution in the section was measured: (**a**) Uniform magnetic field distribution; (**b**) magnetic flux density at each point on the X-axis.

The current flowing into the Helmholtz Coil generates a uniform magnetic field, and the simulation results are shown in Figure 5.

In Figure 5a, the value range of the color bar is between 0.953–1.02 mT. It can be seen that the magnetic field distribution of the whole measured section is relatively uniform, and the red arrow represents the direction of the magnetic field. Figure 5b shows the distribution curve of magnetic flux density at each point on the x axis. The measured regional coordinate interval in this paper is [−0.035,0.035], within which the magnetic flux density value is basically stable at 1 mT.

**Figure 6:**
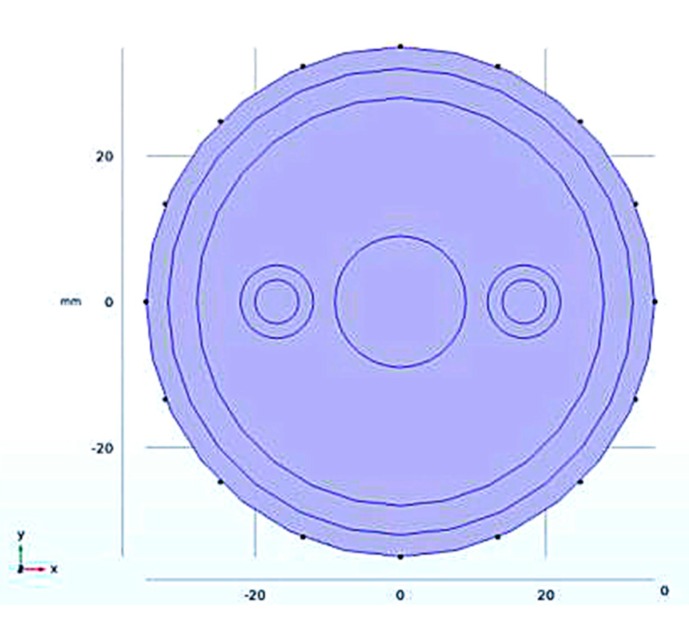
The position of the measured induced potential.

In the simulation study in this paper, the blood flow velocity was set as 0.23 m /s, the conductivity was set as 1.09 S/m [32], and the induction potential was obtained by means of 16-electrode measurement, as shown in Figure 6. According to Figure 3, the electrode e_0_ was taken as the reference potential, denoted as $U_{0}$, and then the potentials of the other measuring electrodes were arranged counterclockwise as $U_{1}$,$U_{2}$...$U_{15}$ and make the difference with $U_{0}$ to get 15 induction potential differences as $\Delta U_{1}$,$\Delta U_{2}$...$\Delta U_{15}$.

### 4.2. Verification of Blood Flow Volume Inversion Model

In order to verify that the electromagnetic induction measurement method is insensitive to the number and location of blood vessels, three main tests were performed:

**Test1:** Blood flow was injected into the right artery alone
**Test2:** Blood flow was both injected into two arteries simultaneously
**Test3:** Blood flow was both injected into two arteries after they were rotated 45° to the Y-axis

**Figure 7:**
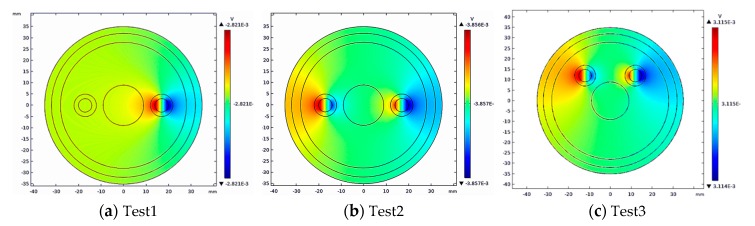
The induction potential distribution of the measured section in different test conditions.

Firstly, the induction potential distribution of the measured section was simulated by COMSOL, as shown in Figure 7.

**Figure 8:**
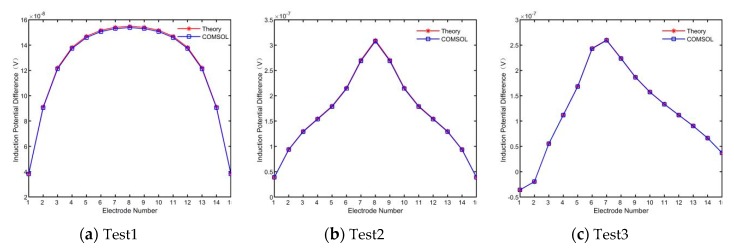
The difference of potential in different test conditions.

Then, 15 groups of induction potential differences were extracted, as shown in Figure 8.

**Figure 9:**
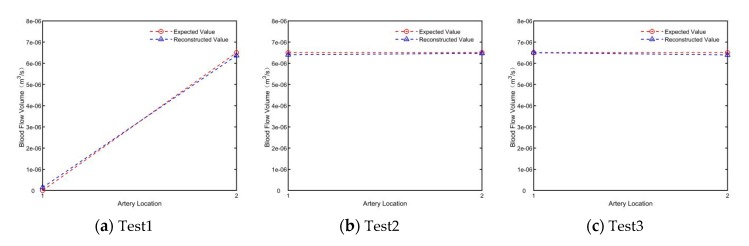
The difference of potential in different test conditions.

According to Figure 8, the induction potential difference obtained in COMSOL is consistent with the theoretical value. The simulated induced potential difference was used for the inverse calculation of blood flow value, and the results are shown in Figure 9:

The results of blood flow reconstruction in the three groups showed that, the blood measurement method based on electromagnetic induction is not sensitive to the number and location of human arteries and blood non-axisymmetric flow, so it can be applied to the blood flow measurement of different individuals, and the error of measurement results is less than 0.1%, which can accurately reconstruct the blood flow information of limb section artery.

### 4.3. Prediction of Arterial Models with Different Rates of Stenosis

**Figure 10:**
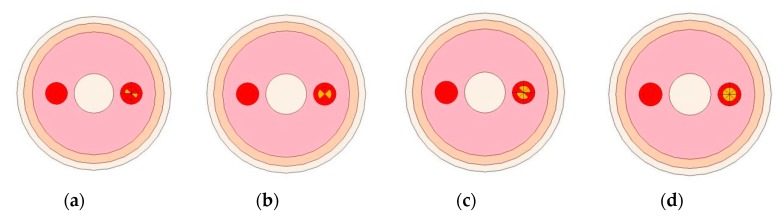
The simulation models of arterial with different rates of stenosis: (**a**) Rate of stenosis: 25%; (**b**) rate of stenosis: 50%; (**c**) rate of stenosis: 75%; and (**d**) rate of stenosis: 100%.

In this section, four typical stenosis artery simulation models were constructed for tests, as shown in Figure 10. The right artery was artery vessels with different rates of stenosis, and the left artery was normal vessels without stenosis, as the reference object.

**Figure 11:**
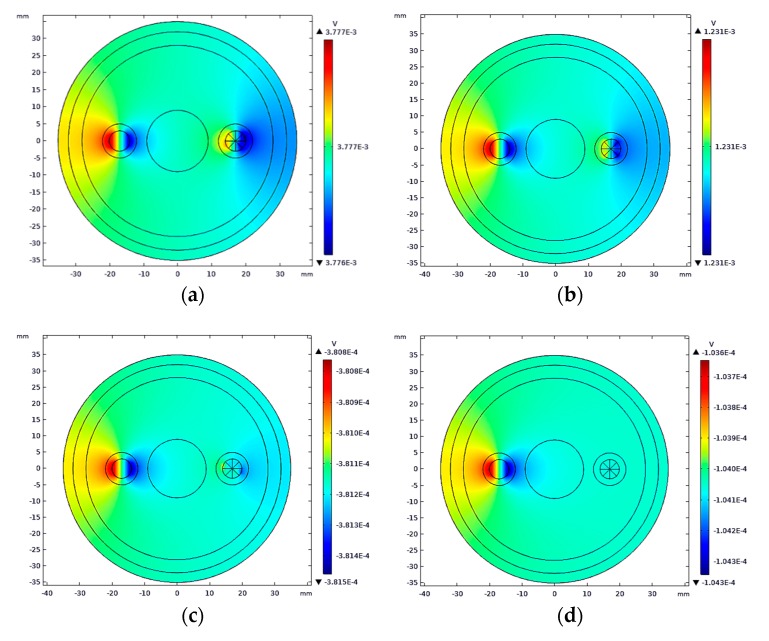
The induction potential profiles of arteries with different rates of stenosis: (**a**) Rate of stenosis: 25%; (**b**) rate of stenosis: 50%; (**c**) rate of stenosis: 75%; and (**d**) rate of stenosis:100%.

The same blood flow was injected into each artery, and the induced potential difference was obtained by COMSOL. The simulation results are shown in Figure 11.

**Figure 12:**
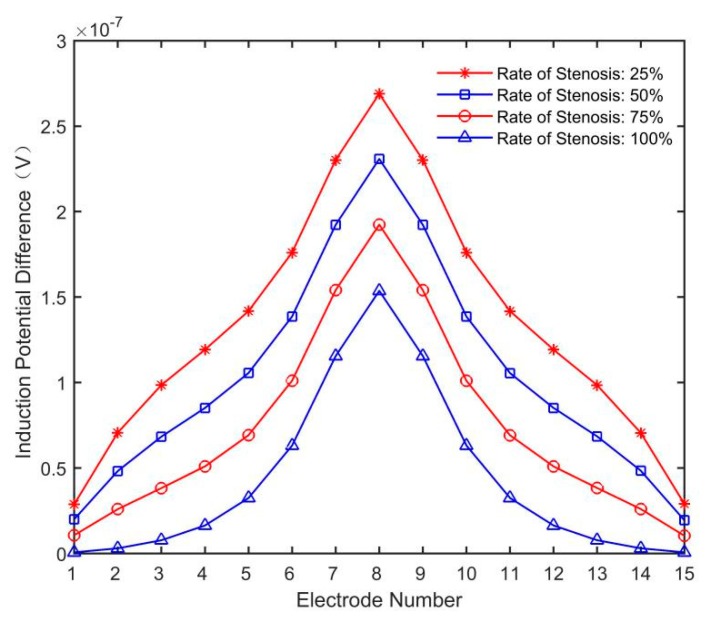
Induction potential difference distribution at different rates of stenosis.

The 15 groups of induction potential differences with U_0_ as the reference potential were extracted, as shown in Figure 12.

**Figure 13:**
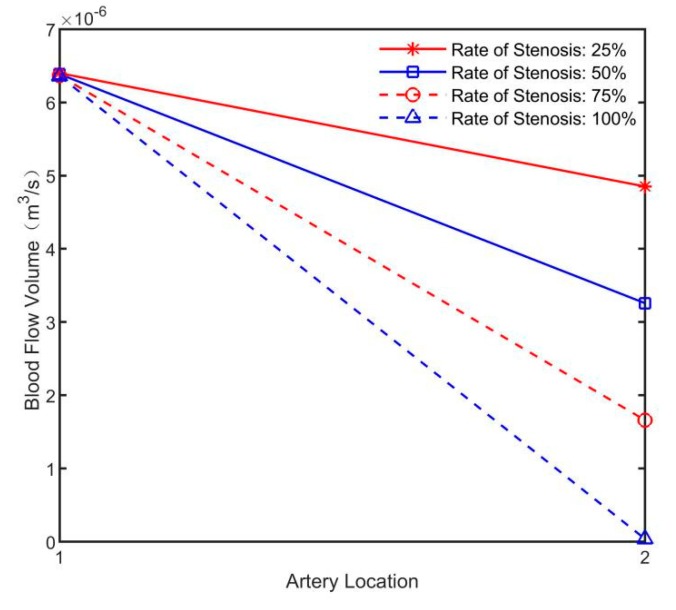
Inverse results of blood flow in arterial models with different rates of stenosis.

As can be seen from Figure 12, the more severe the artery stenosis is, the smaller the amplitude of the induction potential difference will be. The simulated induced potential difference was input into the flow inversion model to obtain the blood flow volume of each artery, as shown in Figure 13.

**Figure 14:**
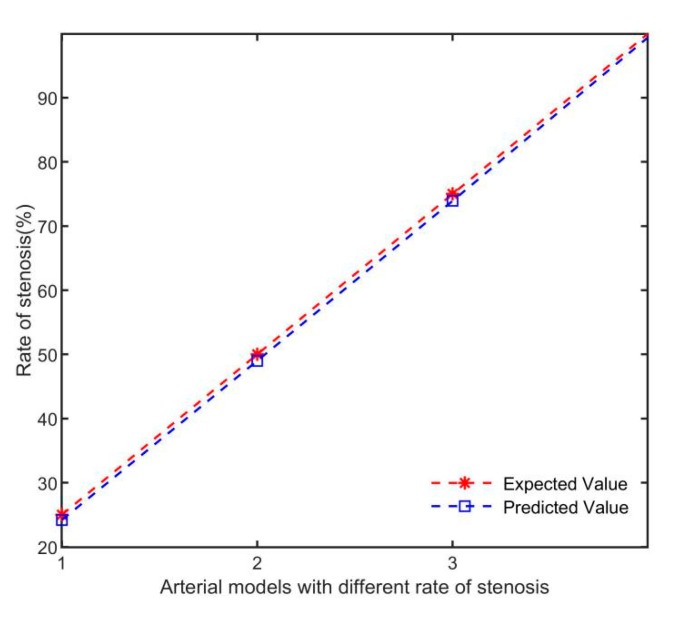
Prediction results of rates of stenosis in different arterial models.

In Figure 13, the normal arterial blood flow values reconstructed by the four simulation models were basically the same, and the arterial blood flow values with different rates of stenosis decreased with the increase of rates of stenosis. The predicted rates of stenosis is shown in Figure 14.

As can be seen from Figure 14, the predicted rates of arterial stenosis has a good consistency with the expected value, and the higher the rate of stenosis, the higher the prediction accuracy. This proves that the blood flow volume linear inversion model based on electromagnetic induction for predicting the rate of arterial stenosis is feasible.

## 5. Conclusions

In this paper, a blood flow volume linear inversion model based on electromagnetic sensor for predicting the rate of arterial stenosis is proposed. According to the electromagnetic induction theory, the blood flow through a uniform magnetic field will generate an inductive potential field. The electromagnetic induction potential signal was obtained from the surface of human skin by the electromagnetic sensor, and the blood flow volume inversion matrix was constructed by combining weight function theory and finite element method, and then the blood flow volume of human artery was calculated. Finally, the rate of stenosis was predicted by the relationship between blood flow and arterial cross section area. The model has been verified by COMSOL and MATLAB simulation, and accurate inversion results of blood flow and prediction results of rates of stenosis have been obtained. The results showed that the blood flow volume linear inversion model based on electromagnetic sensor for predicting the rate of arterial stenosis is feasible. The following work will focus on how to determine the exact location of arterial stenosis, which will be of great significance for the diagnosis and treatment of arterial stenosis.
